# Eine Monotherapie mit Immuncheckpointblockade führt bei Patienten mit Rektumkarzinom und Mikrosatelliteninstabilität zur klinischen Komplettremission

**DOI:** 10.1007/s00066-022-01990-7

**Published:** 2022-08-11

**Authors:** Markus Diefenhardt, Emmanouil Fokas, Claus Rödel

**Affiliations:** Klinik für Strahlentherapie und Onkologie, Universität Frankfurt, Theodor-Stern-Kai 7, 60590 Frankfurt, Deutschland

## Hintergrund

Metastasierte kolorektale Karzinome mit einer Mismatch-Reparatur-Defizienz sprechen auf eine Immuncheckpointblockade bekannterweise gut an. Eine prospektive Phase-2-Studie (ClinicalTrials.gov: NCT04165772) testete den Einsatz des monoklonalen PD-1-Antikörpers („programmed cell death protein 1“) Dostarlimab als systemische Induktionstherapie vor geplanter Radiochemotherapie und Operation bei Patienten mit einem Mismatch-Reparatur-defizienten, lokal fortgeschrittenen Rektumkarzinom.

## Methoden

Dostarlimab wurde als Induktionstherapie über einen Zeitraum von 6 Monaten (alle 3 Wochen, insgesamt 9 Gaben) bei Patienten mit Rektumkarzinom im UICC-Stadium II und III bei nachgewiesener Mismatch-repair-Defizienz (immunhistochemischer Expressionsverlust von MLH1, MSH1, MSH6, PMS2) verabreicht. Anschließend sollten eine Standardradiochemotherapie und eine Operation erfolgen, es sei denn, nach Abschluss der Dostarlimabtherapie wurde ein klinisch vollständiges Ansprechen mittels digital-rektaler Untersuchung (DRU), Endoskopie und MRT des Beckens festgestellt. Als primäre Endpunkte wurden die anhaltende klinische Komplettremission 12 Monate nach Abschluss der Dostarlimabtherapie oder eine histopathologische Komplettremission (pCR) nach Dostarlimabtherapie und Operation mit oder ohne neoadjuvante Radiochemotherapie definiert.

## Ergebnisse

Zum Zeitpunkt der Veröffentlichung hatten 12 Patienten die Behandlung mit Dostarlimab abgeschlossen und wurden für mindestens 6 Monate nachbeobachtet. An Grad-1- und Grad-2-Nebenwirkungen traten Hautrötung (31 %), Pruritus (25 %), und Fatigue (25 %) auf; höhergradige Toxizitäten wurden nicht berichtet. Alle 12 Patienten zeigten beim Restaging nach Induktionstherapie mit dem PD-1-Antikörper eine klinische Komplettremission auf Grundlage der DRU, der Rektoskopie, der MRT, einer FDG-PET und der Biopsie. Kein Patient erhielt daraufhin eine Radiochemotherapie oder eine Operation. Bei einer Nachbeobachtungzeit von 6 bis 25 Monaten (4 Patienten mit mindestens 12 Monaten) ist bislang kein lokales Wiederwachstum des Tumors („local regrowth“) oder eine Metastasierung aufgetreten.

## Schlussfolgerungen der Autoren

Bei Mismatch-Reparatur-defizientem, primärem Rektumkarzinom wurde durch eine Monotherapie mit dem PD-1-Inhibitor Dostarlimab eine hohe Rate an klinischer Komplettremission erreicht. Eine längere Nachbeobachtung ist allerdings erforderlich, um die Dauer des Ansprechens zu beurteilen.

## Kommentar

Die Studie wurde kürzlich beim ASCO 2022 in Chicago vorgestellt und ist trotz geringer Patientenzahl und kurzer Nachbeobachtungszeit bereits im renommierten *New England Journal of Medicine* als Vollpublikation erschienen [1]. Im Vorfeld hatte eine Pilotstudie beim primären, nichtmetastasierten Kolonkarzinom mit Mikrosatelliteninstabilität (MSI-H) gezeigt, dass nach einer neoadjuvanten „Kurzzeit“-Immuntherapie mit nur einer Gabe Ipilimumab und 2 Gaben Nivolumab gefolgt von obligater Operation insgesamt 19 von 20 Patienten eine ausgeprägte Tumorregression aufwiesen (Nachweis von < 10 % residuellen, viablen Tumorzellen; [2]). Beim metastasierten MSI-H-Kolorektalkarzinom ist die Immuntherapie mittlerweile die empfohlene *First-line-*Therapie, nachdem die KEYNOTE-177-Phase-3-Studie bei besserer Verträglichkeit eine signifikante Überlegenheit von Pembrolizumab gegenüber der klassischen Chemotherapie (mFOLFOX6, FOLFIRI) hinsichtlich des progressionsfreien Überlebens gezeigt hat (median 16,5 vs. 8,2 Monate, HR 0,59, 95 %-CI 0,45–0,79; [3]).

Beim Rektumkarzinom gab es zu dieser seltenen molekularen Subgruppe von Patienten (nur ca. 5 % aller Enddarmkarzinome weisen eine MSI‑H auf) bisher nur Einzelfallberichte, die aber schon eine hohe Effektivität der (neoadjuvanten) Immuntherapie nahelegten [4]. Die aktuelle, prospektive Phase-2-Studie testete nun eine auf 6 Monate prolongierte Immuninduktionstherapie mit der Option einer Watch-&-wait-Strategie bei Erreichen einer klinischen Komplettremission (cCR). Während dieser 6 Monate erfolgte ein engmaschiges Monitoring der Tumorantwort mittels repetitiver Endoskopien und Tumorbiopsien, MRT des Beckens und FDG-PET-CT, um einerseits frühzeitig ein fehlendes Ansprechen erkennen zu können und andererseits die spezifische Dynamik der Tumorregression eines rektalen Karzinoms mit MSI‑H unter PD-1-Blockade zu untersuchen.

Interessanterweise wiesen 5 von 10 Patienten in den repetitiven Biopsien bereits 6 Wochen nach Therapiebeginn keine viablen Tumorzellen mehr auf, nach 3 Monaten zeigte die Endoskopie bei 5 von 12 Patienten eine cCR, während die FDG-PET-CT zu diesem Zeitpunkt bereits eine komplette SUV-Reduktion auf Hintergrundlevel aufwies. Demgegenüber entwickelten sich die Response-Kriterien in der MRT-Untersuchung verzögert. In translationalen Begleituntersuchungen war u. a. ein initialer Anstieg der PD-L1-Expression und der Expression von CD8+-T-Lymphozyten in Tumor- und Normalgewebe nach 6 Wochen zu erkennen, begleitet von einem Anstieg von CD20+-B-Lymphozyten als tertiäre lymphoide Strukturen im peritumoralen Stromagewebe.

Warum sind die Komplettremissionsraten der „neoadjuvanten“ Immuntherapie beim MSI-H-Rektumkarzinom so gut und teils deutlich besser als die neoadjuvante Immuntherapie bei anderen soliden Tumoren mit nachgewiesener Sensitivität gegenüber einer Checkpointblockade im metastasierten Setting (z. B. NSCLC, Melanom; [5])? Neben einer adäquaten molekularen Selektion (Expressionsverlust in mindestens 2 der 4 untersuchten Mismatch-Reparatur-Proteine, hohe tumorale Mutationslast) hat wahrscheinlich auch die lange, 6‑monatige Dauer der Immuntherapie zu der hohen Rate an cCR beigetragen. Bekannterweise entwickelt sich nämlich das Tumoransprechen auf eine Immuntherapie verzögert über Wochen bis Monate [6], sodass das mittlerweile beim Rektumkarzinom etablierte lange Intervall von Therapiebeginn bis zur endgültigen Responsebeurteilung (analog den TNT-Regimen bei MSS-Tumoren) mit repetitivem Monitoring der Therapieantwort und selektiven W&W-Optionen eine ideale Studiensituation erlaubte [7].

### Fazit

Für die seltene molekulare Subgruppe von MSI-H-Rektumkarzinomen ist die (alleinige) Immuntherapie eine vielversprechende neue Option, deren Sicherheit und Wirksamkeit natürlich noch bei mehr Patienten über einen längeren Nachbeobachtungszeitraum untersucht werden muss.

*Markus Diefenhardt, Emmanouil Fokas, Claus Rödel, Frankfurt*
